# Preparation and Characterization of Catalase-Loaded Solid Lipid Nanoparticles Protecting Enzyme against Proteolysis

**DOI:** 10.3390/ijms12074282

**Published:** 2011-07-04

**Authors:** Ce Qi, Yan Chen, Qing-Zhe Jing, Xing-Guo Wang

**Affiliations:** 1 School of Food Science and Technology, Jiangnan University, Wuxi 214122, China; E-Mails: jqzwuxi@163.com (Q.-Z.J.); wxg1002@hotmail.com (X.-G.W.); 2 State Key Laboratory of Food Science and Technology, Jiangnan University, Wuxi 214122, China; E-Mail: chenyan8855@163.com; 3 COFCO East Ocean Oils & Grains Industries (Zhangjiagang) Co., Ltd., Zhangjiagang 215634, China

**Keywords:** catalase, solid lipid nanoparticles, proteolysis, enzyme delivery

## Abstract

Catalase-loaded solid lipid nanoparticles (SLNs) were prepared by the double emulsion method (w/o/w) and solvent evaporation techniques, using acetone/methylene chloride (1:1) as an organic solvent, lecithin and triglyceride as oil phase and Poloxmer 188 as a surfactant. The optimized SLN was prepared by lecithin: triglyceride ratio (5%), 20-second + 30-second sonication, and 2% Poloxmer 188. The mean particle size of SLN was 296.0 ± 7.0 nm, polydispersity index range and zeta potential were 0.322–0.354 and −36.4 ± 0.6, respectively, and the encapsulation efficiency reached its maximum of 77.9 ± 1.56. Catalase distributed between the solid lipid and inner aqueous phase and gradually released from Poloxmer coated SLNs up to 20% within 20 h. Catalase-loaded SLN remained at 30% of H_2_O_2_-degrading activity after being incubated with Proteinase K for 24 h, while free catalase lost activity within 1 h.

## 1. Introduction

Active oxygen species such as hydrogen peroxide are readily generated in many cells by metabolic processes such as respiration, ischemia/reperfusion, and oxidation of fatty acids, and they are highly toxic to cells by damaging such components as DNA, lipids, and enzymes. Catalase (CAT, EC 1.11.1.6), an enzyme that reduces hydrogen peroxide to water, is a potential drug to prevent the accumulation of toxic levels of hydrogen peroxide. For example, targeting catalase to endothelial cells lining the blood vessel lumen alleviates vascular oxidative stress in animal models. However, as a therapeutic protein, it does not possess the required physicochemical properties to be absorbed at their target site, or reach or enter the cell. Premature elimination from circulation, as well as inactivation by inhibitors and proteases, limits the effectiveness and utility of this enzyme [1]. Hence, the study of delivery and targeting systems is needed to overcome these disadvantages and improve its performance.

An established strategy for protein delivery consists of attaching the proteins to suitable particulate carrier systems, whereby the *in vivo* fate of the protein molecule is determined by the properties of the carrier system rather than those of the protein [2]. Formulation in solid lipid nanoparticles (SLNs) confers improved protein stability and avoids proteolytic degradation, as well as sustained release of the incorporated protein and seems to fulfill the requirements for an optimum particulate carrier system [3,4].

High-pressure homogenization [5] and microemulsion-based techniques [6] are the most used methods in the preparation of SLN. Double emulsion method (w/o/w), a typical microemulsion-based technique, firstly used for SLN preparation described by Sjöström and Bergenståhl [6], is more moderate and avoids any thermal or pressure stress on the entrapped enzyme [7] when used with the solvent evaporation technique.

This study was aimed to develop and characterize catalase-loaded SLN using the double emulsion method and solvent evaporation technique, in order to obtain a narrow size distribution and a high loading of the biologically active enzyme.

## 2. Results and Discussion

### 2.1. Influence of Organic Solvent Species and Emulsifying Operation on Catalase Activity

Experimental constraints such as sonication and organic solvent might disturb the activity of catalase. Different organic solvents decreased catalase activity to varying extents with acetone/DCM (1:1) causing the lowest loss in activity among the three solvents tested, regardless of whether sonication or vortex was used (Table 1). Therefore, acetone/DCM (1:1) was used as dissolvent of catalase. This was also supported by a study of Gander *et al.* who found that acetone did not disturb the structure of protein [3] and it was often used for the fractionation of plasmatic proteins. The choice of methylene chloride was rational as it has always been used for nanoparticle preparation [8], and served as the solvent for acetone. It was found that susceptibility to the denaturing action of DCM is dependent on the protein type during the primary emulsification step [9]. SDS-PAGE and circular dichroism spectroscopy analysis showed that loading into SLN neither induced catalase fragmentation nor significantly changed in secondary structure (data not shown).

Emulsification was an important step for preparation of SLN. Emulsion from vortexing was found to be less stable than from sonication. However, occurrence of cavitation [10] and increased interaction between enzyme and organic solvent might disturb the enzyme conformation during sonication. Sonication operation was optimized before preparation of SLN. During the two steps of sonication, it was found that activity loss was mainly induced by the first step **(**Table 2**)**. Thus, the sonication time during the second step is preferable to be extended for ample emulsifying effects. So we used the first step of 20 s and a second step of 30 s for emulsifying.

### 2.2. Effect of Lecithin on the Primary w/o Emulsification

In order to improve the entrapment efficiency, amphiphilic lecithin was used to decrease interactions between the aqueous and organic phases. Table 3 shows that lecithin enhanced the stability of the primary w/o emulsion depending on lecithin/triglyceride ratio. The w/o emulsion was very stable when the ratio was more than 5%. Lecithin concentration might determine the thickness of the lecithin layer, which was essential for the stabilization of lipid emulsions [11]. According to the theory of Derjaguin, Landau, Verwey and Overbeek (DLVO), the electrostatic repulsion force and Van der Waals force existing among the particles determine colloidal stability. When nanoparticles were covered by a layer of surfactant, the electrostatic repulsion potential energy among the particles increased then enhanced nanoparticles stability [11].

### 2.3. Production and Characterization of Lipid Nanoparticles

Table 4 shows that volume of outer aqueous phase and lecithin:triglyceride ratio affected the size distribution of the nanoparticles. The size decreased with increase of the lecithin:triglyceride ratio. Increasing lecithin concentrations might favor the creation of additional w/o interfaces, then the smaller particles form. But this tendency no longer continues when the radius of curvature of the interface reaches a particularly low value, this might be due to shaping of other structures like lecithin multilayer. Table 4 shows that when 5% of lecithin/triglyceride was used, polydispersity decreased depending on decreasing of outer aqueous phase volume; in agreement with that reported by Garcıa-Fuentes *et al.* [12]. Two milliliters of outer aqueous phase resulted in lowest polydispersity (0.322–0.354). Smaller outer aqueous phase volumes might receive a higher energy input per gram of coarse emulsion as found in preparation of SLN with high-speed homogenizers [13]. In Table 4, increasing amounts of lecithin resulted in higher polydispersity, due to the possible formation of multiple lecithin layers [14] or other structures such as liposomes [13].

To modify the surface characteristics, Poloxamer 188 or PVA were incorporated in outer aqueous phase during the second emulsification process (Table 5). These surfactants could attach to and coat SLN surface via its hydrophobic segments and increase SLN stability. Table 5 shows that coating with surfactants significantly increased zeta potential of SLN. This might be due to extension of particles near plane [15].

### 2.4. Catalase Loading and Release from the Nanoparticles

Drug loading and releasing properties are key factors determining application efficiency of SLNs. Table 6 shows that Poloxmer 188 coating was better than PVA in increasing encapsulation efficiency, which might reduce the w/o interfacial tension and enhance enzyme-lipid affinity. In general, the lipid matrixes used in SLN resulted in sustained enzyme release profiles, probably due to the nature of the lipid matrix itself and to the affinity of the enzyme to some formulation components [16].

**Figure 1:**
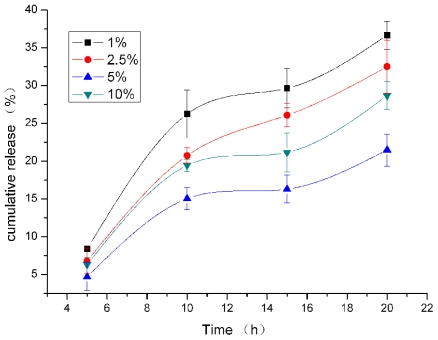
The effect of lecithin:triglyceride ratio on *in vitro* release (mean ± S.D., *n* = 3).

For Poloxmer 188 coated SLN, lecithin:triglyceride ratio greatly influenced catalase releasing (Figure 1). Lowest cumulative release during 20 h was reached when 5% of lecithin:triglyceride ratio was used. Lecithin:triglyceride ratio might change the polymorphic state of the lipid constitutes, inducing a difference in catalase solubility in the lipid matrix, and change drug incorporation [17]. Nevertheless, while the mechanisms controlling drug release of hydrophobic molecules were being elucidated [18], very little work has been carried out on the release of hydrophilic macromomolecules. Almeida *et al*. [19] had achieved a 0.03% (w/w) loading of lysozyme in lipid nanoparticles and Garcıa-Fuentes *et al.* [3,16] achieved >90% loading of calcitonin and cumulative release of about 4% within 6 h. Encapsulation efficiency of catalase in our study reached its maximum of about 77.9% at 20 h, which needs further improvement. Recently, Liu *et al*. have prepared insulin loaded-SLN [20]. They employed sodium cholate and soybean phosphatidylcholine to improve the liposolubility of insulin and reached high entrapment efficiency. Encapsulation efficiency of catalase in SLN might be enhanced by using sodium cholate as co-emulsifier and needs to be studied further.

### 2.5. The TEM Image of Particles Prepared by Different Lipid Matrix

**Figure 2:**
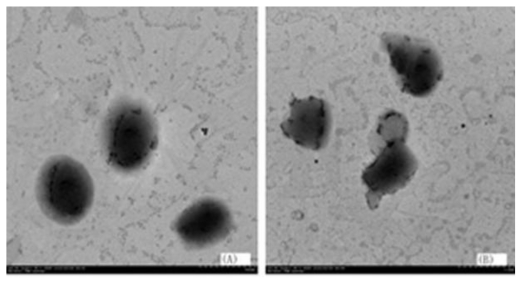
Transmission electron micrograph of catalase-loaded SLN using triglyceride (**A**) or Monoglyceride (**B**) as the lipid matrix. The SLNs were prepared with 4.0 mg catalase, 100.0 mg lipid matrix, 5% lecithin, and 2.0 mL 2% poloxmer 188 of the outer aqueous phase.

As shown in Figure 2, triglyceride based SLN was more round than that of monoglyceride (control). The probable reason was that triglyceride was a nonpolar molecule, but monoglyceride was a polar molecule. When the polar group of the enzyme had contact with the monoglyceride, the molecules of monoglyceride moved and some particles contacted each other, which caused the morphology changing of the particle. In contrast, the morphology of the particle prepared with the triglyceride was more stable, which is why triglyceride is the lipid matrix most commonly used.

### 2.6. The Location of Catalase within SLN

**Figure 3:**
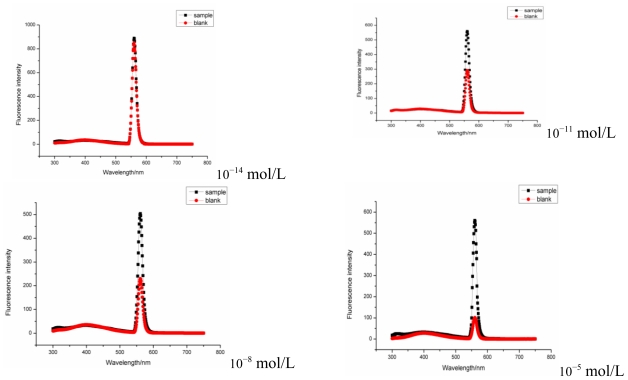
The effect of CuSO_4_ in the outer aqueous phase on the fluorescence intensity of SLN loaded with the same content of catalase. Sample: catalase prepared in SLN. Blank: Catalase added in the outer aqueous phase without SLN. The concentration of CuSO_4_ ranged from 10^−14^ to 10^−5^ mol/L.

Figure 3 shows the fluorescence intensity decreased less in test group than in blank with increasing CuSO_4_ concentration, suggesting that either the outer or inner membranes of the SLNs resist the inward penetration of Cu^2+^, thus limiting the inactivation effects of Cu^2+^ to the catalase residing in the outer phase. Thus we conclude that large amounts of the enzymes in the emulsion are packaged in the inner phase of the SLNs.

**Figure 4:**
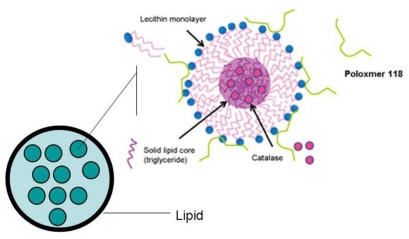
Schematic representation of the structure of catalase loaded SLNs.

Figure 4 is a schematic diagram which represents the proposed structure of catalase loaded SLNs. The long alkyl chains of lipid were twisted with the hydrophobic chains of lecithin which form a continuous shell that became coated with Poloxmer 118. Catalase was located in the inner phase of the SLNs.

### 2.7. Protection of Catalase in Nanocarriers from Proteolysis

**Figure 5:**
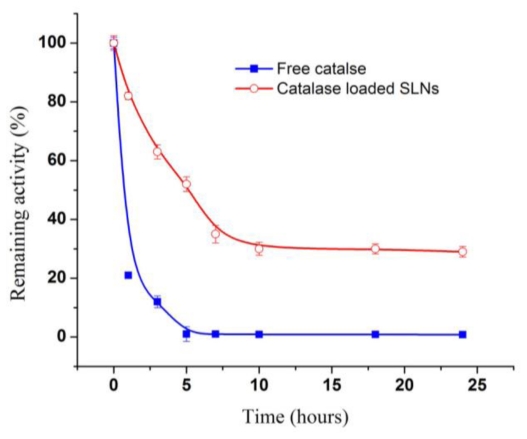
Loss of catalase activity in Proteinase K solution as a function of time (mean ± S.D., *n* = 3).

The time course of the proteolytic loss of catalase activity was determined (Figure 5). Free catalase lost about 70% of its activity after 1 h incubation, and fell below measurable levels at 5 h; in contrast, SLN loaded catalase retained 48% of its initial activity at 5 h, and reached a plateau corresponding to 30% of its initial activity and remained at a stable level for at least ~24 h. The loss of activity in the SLN is believed to be associated with the catalase that is either surface bound or is released into the aqueous medium. These results indicate that catalase loaded in SLN was protected against proteolysis, yet was still capable of degrading H_2_O_2_ diffusing through the polymer shell. Dziubla *et al*. have loaded catalase into polymer nanocarriers containing poly (ethylene glycol) -*b*-poly (lactic-glycolic acid) (PEG- PLGA) [21]. Catalase in nanocarriers stably retained 25–30% of H_2_O_2_-degrading activity for at least 18 h in a proteolytic environment. Poloxmer 188 coating and lipid matrix in catalase-loaded SLN might be more effective to prevent protease diffusion in nanoparticles than PEG-PLGA.

## 3. Experimental

### 3.1. Materials

Catalase Assay Kit was purchased from Nanjing Jiancheng Institute of Biology (Nanjing China). Poloxmer 188 was purchased from BASF (Germany). Poly (vinyl alcohol) (PVA) of 13000–23000 g/mol (87–89% hydrolysed) was supplied by Sigma-Aldrich (St. Louis, MO, USA). Lecithin was purchased from Cargill Company (Nantong, China). Catalase was purchased from Shanghai Kayon Biological technology Co., Ltd (Shanghai, China). Monoglyceride and Triglyceride (tripalmitin) were offered by Wilmar Co., Ltd (Shanghai, China). Other chemicals and solvents were obtained from Chinese Chemical Company (Shanghai, China) and were of A.R. grade.

### 3.2. Determination of the Effect of Organic Solvents on Catalase Activity

A 0.2 mL catalase solution (0.2 g/L in PBS, pH 7.0) was treated with sonication or vortexing, succeeded by re-sonication for different time following PBS (pH 7.0) adding to a final volume of 2.0 mL.

### 3.3. Determination of Catalase Activity

Catalase activity was determined with Catalase Assay Kit. The reaction of H_2_O_2_ decomposition by catalase was terminated by adding ammonium molybdate, with the remaining H_2_O_2_ and ammonium molybdate producing a pale yellow complex which was measured at 405 nm. One enzyme unit was defined as 1.0 μmol H_2_O_2_ decomposed by one milligram catalase per minute at pH 7.0 and 37 °C. All assays were carried out in triplicate.

### 3.4. Preparation of Catalase-Loaded Nanoparticles

Double emulsion method (w/o/w) and solvent evaporation techniques [4, 14] was used. Briefly, 0.20 mL catalase solution (20.0 mg/mL in PBS, pH 7.0) (aqueous phase) was emulsified in 1.0 mL organic solvent containing 100.0 mg triglyceride and different amounts of lecithin (oily phase). This mixture was ultrasonically dispersed in ice bath for desired seconds at 20 W or treated by vortexing to produce a w/o emulsion. Then, different volumes of a 2% (w/v) Poloxamer 188 or PVA solution, as the outer aqueous phase, were added and sonicated for desired seconds. The organic solvent was evaporated by rotary evaporation. Nanoparticles were recovered by centrifugation at 12 000 × g for 30 min at 4 °C.

### 3.5. Evaluation of the Stability of the Emulsion

A stable opaque and opalescent emulsion was obtained during an effective emulsification processing. It was regarded as an un-emulsified product, in which the oil and water separated quickly after sonication. Emulsion stability was evaluated by monitoring its stable time. When the emulsion was kept still at room temperature, the time during which phase separation was not detected was regarded as the stable time.

### 3.6. Determination of Particle size and Zeta Potential

Particle size distribution (mean diameter and polydispersity index) and zeta potential was determined on a Zetasizer Nano (Malvern Nano-ZS 90, England) station. Each nanoparticle preparation was analyzed in distilled water at a concentration recommended by the manufacturer of the instrument.

### 3.7. Determination of Encapsulation Efficiency

The encapsulation efficiency (*EE*) is defined as the percentage of enzyme entrapped in the vesicles in relation to the total amount of enzyme present during the vesicle formation and entrapment procedure. Catalase in the nanoparticles was determined indirectly by measuring the amount of free catalase in the supernatant after evaporation of the organic solvent and centrifugation. The concentration of catalase in the aqueous phase was determined by Bradford Assay with bovine serum albumin as a standard. The EE was calculated by the following Equation:

$$EE(\%)=\frac{wt.\;of\;CAT\;used-wt.\;of\;free\;CAT}{wt.\;of\;CAT\;used}$$

### 3.8. Transmission Electron Microscopy (TEM)

The morphology of the particles was examined by TEM (Hitachi H-9500, Japan) after staining with 0.1 mol/L osmium tetraoxide.

### 3.9. *In Vitro* Release Assay

*In vitro* release was performed by suspending 40.0 mg enzyme-loaded nanoparticles in 2.0 mL phosphate buffer (pH 7.4), equilibrated at 37 °C with shaking at 100 rpm in an incubator (THZD; Taicang Laboratory Equipment Factory, China). At fixed time intervals, 1.0 mL suspension was transferred to a 2.0 mL microtube and centrifuged at 12 000 × g (AnkeTGL-16B; Shanghai Anting Scientific Equipment Factory, China) for 20 min. The supernatant was taken for enzyme quantization. The precipitate was suspended with 1.0 mL fresh phosphate buffer and transferred back to the original tube for further release studies.

### 3.10. Determination of the Distribution of Catalase in SLNs

There are three models of incorporation of active compounds into SLN [22], namely, homogeneous matrix, compound free-core with compound enriched outer shell, and drug-enriched core with lipid shell. The fluorescence spectrum was used to detect the location of catalase in SLN with excitation wavelength at 280 nm. Catalase in the outer phase serve as the blank and different concentration of CuSO_4_ was added as the denaturing agent in the outer phase.

### 3.11. Determination of the Proteolysis Resistant Effect

Catalase loaded SLNs were incubated at 37 °C in a pH7.3 PBS solution containing 0.2% proteinase K (w/v). Aliquots were taken at specific intervals of incubation and measured for either enzymatic activity or protein load.

## 4. Conclusions

These preliminary studies indicate that catalase can indeed be loaded with lipid nanoparticles and particle coating with Poloxmer 188 increased the stability of the nanoparticles. Under the same conditions, the uncoated lipid nanoparticles suffered aggregation and 53% degradation in 20 h, thus showing that coating enables oral administration. Loading in SLN protected catalase from proteolysis significantly. However, more studies need to be performed in order to define the appropriate conditions to further optimize the release behavior of lipid nanoparticles.

## Figures and Tables

**Table 1:** Effect of the organic solvent and the sonication time on the catalase activity (mean ± S.D., *n* = 3).

*Emulsifying Operation*	Organic Solvent	Activity of Catalase
Sonication	Ethyl acetate	35.16 ± 0.6
10 s + 10 s	Methylene chloride	57.62 ± 2.39
	Acetone/methylene chloride (1/1)	62.28 ± 1.8

Vortex	Ethyl acetate	42.36 ± 1.19
10 s + 10 s	Methylene chloride	69.48 ± 2.4
	Acetone/methylene chloride (1/1)	73.3 ± 1.8

**Table 2:** Effect of the sonication time on the catalase activity (mean ± S.D., *n* = 3).

*Emulsifying Operation*		Activity of Catalase
no treatment		100

Sonication	30 s + 30 s (pulsed)	43.56 ± 3.59
	20 s + 20 s (pulsed)	55.68 ± 4.19
	10s + 10 s (pulsed)	61.35 ± 3.59

Vortex	10s + 10 s (pulsed)	76.26 ± 2.4
	10s + 30 s (pulsed)	60.42 ± 1.8

**Table 3:** Stable time (min) of w/o emulsion (mean ± S.D., *n* = 3).

Lecithin: Triglyceride (%, w/w)	Stable Time (min)
1	33.67 ± 1.15
2.5	58.33 ± 0.58
5	97.33 ± 2.52
10	106.33 ± 1.53

**Table 4:** Influence of some technological conditions (volume of the outer aqueous phase and lecithin concentration) on the particle size and polydispersity of lipid nanoparticles (mean ± S.D., *n* = 3).

Volume of the Outer Aqueous phase (mL)	Lecithin: TriglyCeride (%,w/w)	Size (nm)	Polydispersity Index Range
5	5	308 ± 8.4	0.474–0.545
3	5	316 ± 4.8	0.382–0.410
2	10	305 ± 4.0	0.422–0.432
2	5	296.0 ± 7.0	0.322–0.354
2	2.5	343.0 ± 9.9	0.270–0.289
2	1	366.5 ± 7.8	0.248–0.268

**Table 5:** Physicochemical properties of surface modified SLN (mean ± S.D., *n* = 3).

Lecithin: Triglyceride (%)	Surfactant in Outer Aqueous Phase (%)	Size (nm)	Polydispersity Index Range	Zeta Potential (mV)
2.5	Poloxamer 188 (2%)	284.8 ± 13.7	0.262–0.273	−39.0 ± 0.4
5%	Poloxamer 188 (2%)	296.0 ± 7.0	0.322–0.354	−36.4 ± 0.6
2.5%	PVA (2%)	277.2 ± 6.6	0.249–0.308	−40.4 ± 0.7
2.5%	–	–	–	−14.1 ± 0.4

**Table 6:** Effect of outer coating on the encapsulation efficiency (mean ± S.D., *n* = 3).

Surfactant in Outer Aqueous Phase	Encapsulation Efficiency (%)
Poloxmer 188	77.9 ± 1.56
PVA	61.36 ± 0.86
No coating (control)	53.90 ± 1.85
